# Are eyes special? Gaze, but not pointing gestures, elicits a reversed congruency effect in a spatial Stroop task

**DOI:** 10.3758/s13414-023-02774-6

**Published:** 2023-08-16

**Authors:** Mario Dalmaso, Giovanni Galfano, Luigi Castelli

**Affiliations:** Department of Developmental and Social Psychology, University of Padova, Padova, Italy

**Keywords:** Eye gaze, Arrow, Pointing gestures, Attention, Spatial-Stroop task

## Abstract

Gaze stimuli can shape attention in a peculiar way as compared to non-social stimuli. For instance, in a spatial Stroop task, gaze stimuli elicit a reversed congruency effect (i.e., faster responses on incongruent than on congruent trials) as compared to arrows, for which a standard congruency effect emerges. Here, we tested whether the reversed congruency effect observed for gaze can emerge for other social signals such as pointing gestures. Participants discriminated the direction (left or right) indicated by gaze and pointing finger stimuli that appeared leftwards or rightwards with respect to a central fixation spot. Arrows were also employed as control non-social stimuli. A reversed congruency effect emerged for the gaze, whereas a standard congruency effect emerged for both the pointing finger and the arrows. This suggests that the reversed congruency effect is specific to gaze stimuli and does not embrace all social signals conveying spatial information.

## Introduction

Gaze stimuli can deeply shape visual attention (e.g., Capozzi & Ristic, 2018; Dalmaso et al., 2020a). One of the most investigated phenomena is the tendency to orient visual attention towards the same spatial location gazed at by others (a form of social attention known as ‘gaze cueing’; e.g., Friesen & Kingstone, 1998). At the behavioural level, several studies reported that, in healthy participants, the magnitude of gaze cueing can be virtually identical to the cueing effect elicited by arrow stimuli, which are non-social stimuli known to induce attentional shifts in an observer (e.g., Dalmaso et al., 2020b; Hermens & Walker, 2010; Kuhn & Kingstone, 2009). For some authors, the strong similarities between gaze and arrow cueing would reflect a domain-general mechanism involved in the processing of the two spatial cues (e.g., Callejas et al., 2014).

In recent years, different tasks have been proposed to investigate whether dissociations between gaze and arrow can emerge. One approach is to explore the processing of gaze and arrow stimuli when they are targets rather than cues for spatial attention, as in the case of a spatial Stroop task in which participants are asked to discriminate the direction indicated by arrow or eye-gaze stimuli (Cañadas & Lupiáñez, 2012). The stimulus (either arrow or gaze) can appear to the left or right of a central fixation point, although its spatial location is task-irrelevant. The typical results show that when the direction indicated by the arrow (e.g., left) is congruent with its spatial location (left), responses are faster than when it appears on the opposite location (right), consistent with the classic results reported in spatial Stroop tasks (see Lu & Proctor, 1995). However, this standard congruency effect (SCE) is generally reversed for gaze stimuli, with faster responses emerging when the direction indicated by the gaze is incongruent, rather than congruent, with its spatial location. This reversed congruency effect (RCE) appears as a robust phenomenon, replicated under different experimental conditions and by different groups (e.g., Chacón-Candia et al., 2020; Hemmerich et al., 2022; Ishikawa et al., 2021; Jones, 2015; Tanaka et al., 2023). Nevertheless, the origins of these effects are still debated. According to Marotta et al. (2018), possible explanations call into question different – yet complementary – mechanisms involved in social attention such as, for instance, ‘*eye contact*’: on incongruent trials, gaze stimuli would be perceived as actually looking at the participants, thus becoming particularly relevant signals that are able to capture attention more strongly than gaze stimuli looking elsewhere. Another explanation discussed by Marotta et al. (2018) relies on ‘*joint attention*’, which refers to the situation where two individuals are looking towards the same object. In this regard, attention would be oriented more strongly towards faces that establish an episode of joint attention with an observer rather than towards faces looking elsewhere (Edwards et al., 2015). In the spatial Stroop task, on incongruent trials, the peripheral stimulus is pointing towards the object to which participants’ attention is expected to be allocated (i.e., the fixation cross). This could explain why the RCE emerges for gaze but not for arrow stimuli, which, due to their symbolic nature, lack any social intentionality. In other words, as for the specific case of gaze stimuli, an incongruent trial creates a context in which *someone else* is looking towards the same location attended by the observer. That is, by definition, an episode of *joint attention* between two individuals (which does not take place in the case of arrows) that would cause an attentional prioritisation towards the joint gaze. More recently, Hemmerich et al. (2022) have proposed a joint distraction account according to which, on congruent trials, eye-gaze stimuli would be unique in their ability to withdraw attention from the relevant task area, thus increasing response times.

The social nature of the RCE has been also supported by the observation that such a phenomenon is influenced by facial expressions (e.g., Jones, 2015; Torres-Marín et al., 2017) and, unlike arrows, by individual social anxiety (Ishikawa et al., 2021). Critically, the RCE does not occur when non-social stimuli resembling faces are used and participants are asked to appraise them as social targets (Cañadas & Lupiáñez, 2012). However, eye gaze is not the only social signal communicating spatial meaning. In everyday interactions, humans make extensive use of pointing gestures, such as when they use the index finger to signal relevant objects in the environment. Indeed, pointing gestures are essential in both social development and communicative skills (e.g., Matthews et al., 2012), and can also orient attention in an observer (Ariga & Watanabe, 2009; Gregory & Hodgson, 2012). Moreover, like arrows, in healthy participants the spatial shifts of attention elicited by pointing gestures appear to be quantitatively similar to those elicited by eye-gaze stimuli (e.g., Cazzato et al., 2012; Dalmaso et al., 2015). Hence, pointing gestures can be considered reliable communicative social signals that can be used in conjunction with (or alternatively to) eye gaze to establish joint attention with others.

In the present study, we explored whether the RCE for gaze emerging from the spatial Stroop task can also be detected for pointing gestures, with the aim of testing whether the RCE is specific for eye-gaze stimuli or it extends to other social signals. We developed a modified version of the spatial Stroop task proposed by Marotta et al. (2018) in which three different target stimuli were used: Arrow, eye gaze and pointing finger. For the arrow stimulus we expected to observe the SCE, whereas for the eye-gaze stimulus we expected to observe the RCE, consistent with previous studies (e.g., Marotta et al., 2018). Most relevant is the analysis of the effect related to pointing finger stimuli. In this regard, two opposite hypotheses can be proposed. On the one hand, the presence of the RCE, like that expected for the gaze, would support the view that pointing fingers and eye gaze are processed similarly, as they both represent social cues that are informative about the allocation of attention of another individual. On the other hand, the presence of the SCE for pointing fingers (similar to that expected for arrows) would be consistent with the notion that not all social stimuli conveying a spatial meaning are processed similarly and that eye gaze is unique in shaping attentional responses.

## Methods

### Participants

The sample size was determined by considering the guidelines (Brysbaert & Stevens, 2018) suggested for linear mixed-effect models (see Results section): A minimum of 1,600 observations per experimental cell should be collected. Given our experimental design, the minimum required sample size was about 67 participants. Students at the University of Padova participated on a voluntary basis. Data collection was carried out online and was closed after about 1 week in which no new respondents were recorded, once verified that the minimum number of participants had been met. The final sample consisted of 192 participants (mean age = 25 years, *SD* = 9.18, 52 males, 16 left-handed). All participants signed an informed consent form. The study was conducted in accordance with the guidelines laid down in the Declaration of Helsinki.

### Procedure

**Fig. 1:**
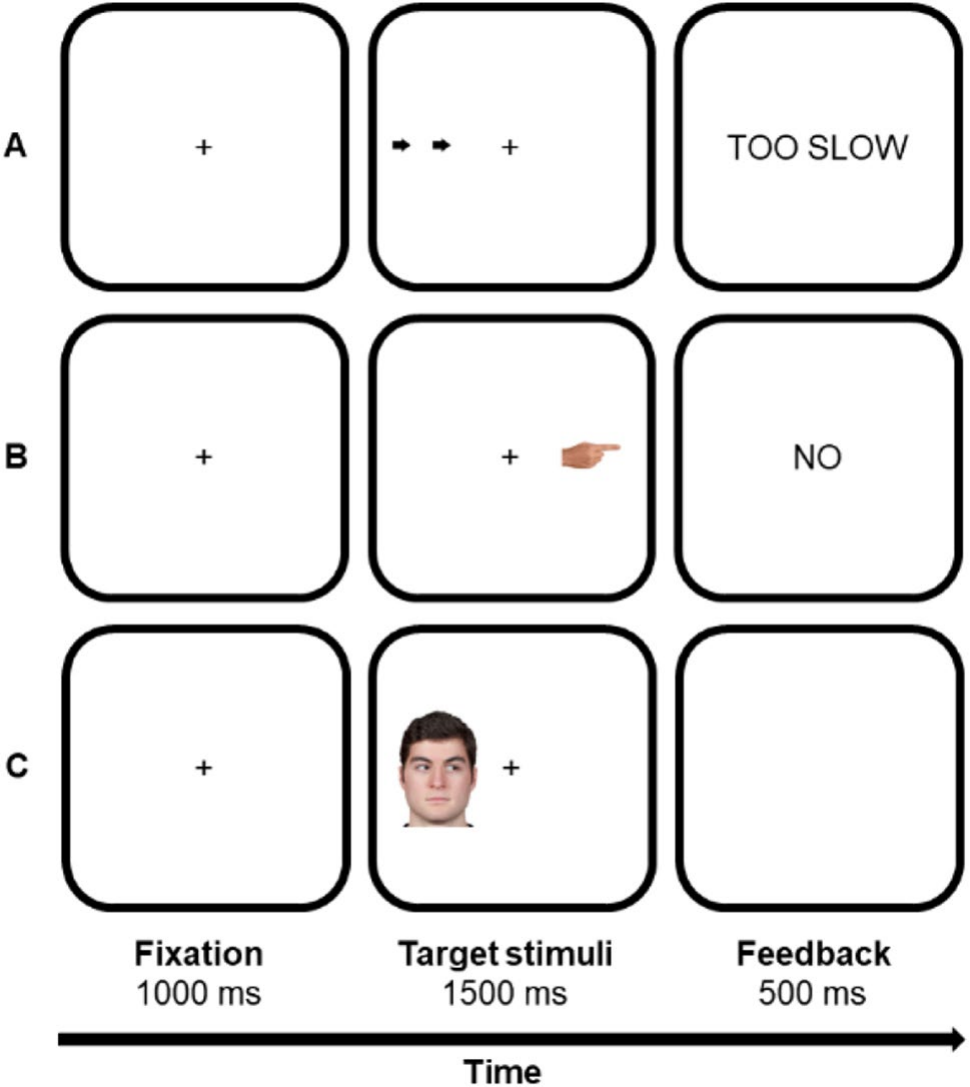
Examples of trials and stimuli (not drawn to scale). Arrows (**A**), a pointing finger (**B**), or a face with averted gaze (**C**) appeared either leftwards or rightwards with respect to the fixation cross. Participants classified the direction (left or right) in which the stimulus was pointing at. Each target type was presented in a different block of trials. In all blocks, visual feedback was provided in case of slow/incorrect responses, whereas correct responses were followed by a blank screen

The experiment was programmed with PsychoPy and delivered online with Pavlovia, which guarantees reliable data (Bridges et al., 2020). Each trial started with a central black fixation cross (Arial font, 0.1 of normalised units), on a white background, lasting 1,000 ms (see Fig. 1). Then, the target stimulus could appear either leftwards or rightwards with respect to the central cross (± 0.2 of normalised units; timeout: 1,500 ms). Three targets were used: The face of a young adult male, with a neutral expression and gaze averted leftwards or rightwards (about 300 px width × 480 px height), a hand with the index finger pointing leftwards or rightwards (about 170 px width × 80 px height), and two black arrows both pointing leftwards or rightwards (each arrow was about 40 px width × 50 px height). For all targets, the parts conveying spatial information (e.g., eye gaze for the facial stimulus) were roughly the same size. The face stimulus was extracted from the standardised MR2 face database (Strohminger et al., 2016; see also Dalmaso et al., 2021). A single face was used because only one type of arrow and pointing finger stimuli were also utilised. Participants were asked to classify, as quickly and accurately as possible, the direction in which the target was pointing at by pressing the ‘F’ key (with the left index finger) for ‘left’ and the ‘K’ key (with the right index finger) for ‘right’. They were also asked to keep their eyes at fixation and to ignore the location (left or right) in which the target could appear, as it was task-irrelevant. Missing and incorrect responses were signalled by a 500-ms visual feedback (the words ‘TOO SLOW’ and ‘NO’, respectively) and correct responses were followed by a 500-ms blank screen. The three targets were presented in three distinct blocks selected in random order. Each experimental block included 48 trials and was preceded by eight practice trials.

## Results

Trials with missing and incorrect responses (0.5% and 2.9% of total trials, respectively) were discarded.^1^ Correct trials with a latency shorter or longer than 3 SDs of each participant’s mean (calculated separately for each experimental condition) were considered outliers and discarded (0.97% of total trials). After data cleaning, there were, for each experimental condition, a minimum of 4,281 observations, and therefore statistical power was adequate (Brysbaert & Stevens, 2018).

**Table 1:** Percentage of correct responses (and SEM) observed in all experimental conditions

	Arrow	Pointing finger	Gaze
Congruent	98.35% (0.001)	98.56% (0.001)	98.97% (0.001)
Incongruent	94.66% (0.003)	95.34% (0.003)	96.63% (0.002)

**Fig. 2:**
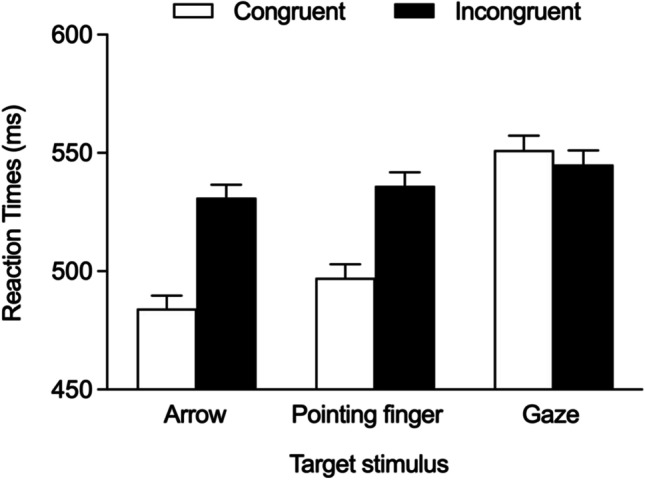
Mean reaction times (and SEM) observed for congruent and incongruent trials as a function of Target stimulus

Data were analysed through a linear mixed-effects model by using the R package ‘*lme4*’ (Bates et al., 2015). Fixed effects were Congruency, Target stimulus, and their interaction. Random effects were the intercepts for participants and the by-participant random slopes for the effects of Congruency and Target stimulus. This was the model associated with the best fitting data established by a likelihood ratio test, which compared different models with an increasing level of complexity (i.e., from the null to the saturated model). A Type 1 ANOVA (implementing Satterthwaite’s approximation for degrees of freedom) for linear mixed-effects models was then used to analyse the model. Effect sizes were calculated by using a standard procedure to get a more direct comparison with the previous works employing this task. When necessary, paired comparisons (Tukey’s HSD) for linear mixed-effects models were computed. Congruency yielded a significant effect, *F*(1, 187.7) = 154.972, *p* < .001, *η*^*2*^_*p*_ = .442, due to shorter RTs on congruent trials (*M* = 511 ms, *SE* = 5.13) than on incongruent trials (*M* = 538 ms, *SE* = 5.11). Target stimulus yielded a significant effect, *F*(2, 189.2) = 36.672, *p* < .001, *η*^*2*^_*p*_ = .178, due to similar RTs (*p* = .08) for arrows (*M* = 508 ms, *SE* = 5.41) and pointing fingers (*M* = 517 ms, *SE* = 5.67), while RTs for gaze were larger (*M* = 548 ms, *SE* = 5.97) than for any of the other two stimuli (*p*s < .001). The slower responses to eye-gaze stimuli are consistent with previous works (e.g., Marotta et al., 2018, 2019; Román-Caballero et al., 2021a), and could reflect the presence of social content as well as the greater perceptual complexity associated with eye-gaze stimuli. Importantly, the Congruency × Target stimulus interaction was also significant, *F*(1, 25684.7) = 207.374, *p* < .001, *η*^*2*^_*p*_ = .387. The paired comparisons showed that RTs were shorter on congruent trials than on incongruent trials for both the arrow target (*M*_*cong*_ = 484 ms, *SE* = 5.55; *M*_*incong*_ = 531 ms, *SE* = 5.61, *p* < .001, *d* = -1.097) and the pointing finger target (*M*_*cong*_ = 497 ms, *SE* = 5.83; *M*_*incong*_ = 536 ms, *SE* = 5.82, *p* < .001, *d* = -.919), whereas the reversed pattern emerged for the eye-gaze target, with RTs being shorter on incongruent trials (*M* = 545 ms, *SE* = 6.07) than on congruent trials (*M* = 551 ms, *SE* = 6.16, *p* = .022, *d* = .142; see also Fig. 2).

## General discussion

A spatial Stroop task was used to investigate whether pointing gestures would elicit the RCE documented for eye-gaze stimuli or, instead, the SCE similar to that observed for arrows. Participants were asked to discriminate the direction of eye gaze and pointing finger stimuli appearing leftwards or rightwards with respect to a central fixation cross. The spatial location of the stimuli was irrelevant to the task. Arrow stimuli were also used as a control, non-social condition. Consistent with previous studies (e.g., Hemmerich et al., 2022; Marotta et al., 2018), the RCE emerged for gaze stimuli whereas the SCE emerged for arrows. Importantly, pointing finger elicited the SCE. The robustness of the observed pattern has been further supported by a recent study addressing the effects elicited by pointing gestures in a spatial Stroop task (Bonventre & Marotta, 2023). Taken together, these results confirm the peculiarities of eye gaze in shaping visuo-perceptual mechanisms in this task. The observation that pointing fingers and arrows elicited similar effects provides additional insight into the comprehension of the RCE elicited by eye gaze. Indeed, it is important to recall that when arrows, pointing gestures and eye-gaze stimuli are used in tasks designed to study spatial cueing of attention, they generally lead to similar results in healthy participants (e.g., Cazzato et al., 2012; Dalmaso et al., 2015). One possibility is that using these stimuli as targets rather than accessory, task-irrelevant, spatial cues resulted in increasing the likelihood of a full processing of the three types of items, which in turn resulted in different behavioural results. More specifically, spatial cueing paradigms often rely on the explicit instruction to ignore cue stimuli, whereas in the spatial Stroop task the stimuli themselves provide the information for selecting the appropriate response.

Pointing gestures are social stimuli in that, similar to eye gaze (and unlike arrows), they typically belong to intentional agents. From a pointing finger, we may rapidly derive information about where another individual wants us to allocate our attention. Despite the strong social nature of this communicative cue, it is noteworthy that the observed pattern of findings paralleled the one that emerged in the case of arrows, further suggesting the likely uniqueness of eye gaze. One possibility is that, unlike eye gaze, pointing gestures and arrows may both serve a prescriptive function. Hence, while eye gaze provides information about the location presumably attended by others (with no explicit request to orient our attention accordingly), pointing gestures and arrows more typically signal the location where we are somehow ‘pushed’ to shift our attention. This common feature may somehow overrule the social meaning of pointing gestures and play a role in the observed differences with respect to eye stimuli in the behavioural data. An alternative possibility is that the different pattern concerning pointing gestures and eye gaze may in part reflect low-level differences in processing perceptual features of the two stimuli (e.g., figure-background segregation; Román-Caballero et al., 2021a, b). Because the empirical evidence is mixed and there are indeed findings not entirely consistent with this view (Cañadas & Lupiáñez, 2012), future research will need to address this issue more directly.

The notion that eye-gaze stimuli can shape visuo-attentional mechanisms peculiarly has been confirmed by a large bulk of evidence at both the ontogenetic and the phylogenetic levels. For example, humans appear to be already equipped at birth with specific mechanisms devoted to detect geometrical patterns resembling eye gaze (e.g., Reid et al., 2017). Sensitivity to eye-gaze stimuli can also be detected in several animal species, while the sensitivity to pointing gestures, although it can still be observed, is less evident and unambiguous (see Shepherd, 2010).

The relevance of eye-gaze stimuli over pointing gestures appears to be also confirmed by converging neuroimaging evidence, indicating the presence of a widespread neural architecture devoted to eye-gaze processing (e.g., Stephenson et al., 2021). In particular, the superior temporal sulcus (STS) appears as the brain area dedicated to the elaboration of the changeable aspects of faces, such as, precisely, eye-gaze direction, although there is also evidence reporting activation of STS even in response to pointing gestures (Sato et al., 2009). Unfortunately, the neural underpinnings of the congruency effect emerging from the spatial Stroop task with social stimuli have been little explored. In this regard, the only work conducted so far recorded electrophysiological measures (ERPs) in response to arrow and gaze stimuli and showed that, while a common interference modulation would emerge for gaze and arrow stimuli early after target onset, opposite conflict effects would be detectable at later stages of processing (Marotta et al., 2019). Future works based on neuroimaging techniques (e.g., fMRI) could hopefully provide new insights to better qualify the results emerging from the spatial Stroop task employed here.

Another avenue for future work would be the idea of employing the spatial Stroop task in specific populations characterized by atypical responses to eye gaze (e.g., attention-deficit hyperactivity disorder (ADHD); see, e.g., Marotta et al., 2013) or pointing gestures (e.g., anorexia nervosa; see, e.g., Dalmaso et al., 2015), to test the generalizability of the RCE for gaze also in clinical contexts.

To conclude, this work confirmed the presence of the RCE for eye gaze in a spatial Stroop task, which appears not to embrace other social stimuli conveying a spatial meaning, such as pointing gestures. This speaks in favour of the possible uniqueness of gaze for the cognitive mechanisms that support social interactions.

## Footnotes

1 Although accuracy analyses are not particularly informative here given the very low percentage of errors, no evidence for a speed-accuracy tradeoff emerged in the data (see also Table 1).
